# Hepatitis B virus infection and the risk of gastrointestinal cancers among Chinese population: A prospective cohort study

**DOI:** 10.1002/ijc.33891

**Published:** 2021-12-14

**Authors:** Tong Liu, Chunhua Song, Youcheng Zhang, Sarah Tan Siyin, Qi Zhang, Mengmeng Song, Liying Cao, Hanping Shi

**Affiliations:** ^1^ Department of Gastrointestinal Surgery Capital Medical University Affiliated Beijing Shijitan Hospital Beijing China; ^2^ Department of Clinical Nutrition Capital Medical University Affiliated Beijing Shijitan Hospital Beijing China; ^3^ Beijing International Science and Technology Cooperation Base for Cancer Metabolism and Nutrition Beijing China; ^4^ Key Laboratory of Cancer FSMP for State Market Regulation Beijing China; ^5^ Department of Epidemiology and Statistics, College of Public Health Zhengzhou University Zhengzhou China; ^6^ Department of Hepatobiliary Surgery The People's Hospital of Liaoning Province Shenyang China; ^7^ Department of Graduate School Dalian Medical University Dalian China; ^8^ Department of General Surgery Beijing Children's Hospital, National Center for Children's Health Beijing China; ^9^ Department of Hepatological Surgery Kailuan General Hospital Tangshan China

**Keywords:** cohort, competing risk models, gastrointestinal cancer, hepatitis B virus, incidence

## Abstract

Our study aims to explore the relationship between chronic hepatitis B virus (HBV) infection and the risk of gastrointestinal (GI) cancers including liver, gastric, gallbladder or extrahepatic bile duct, pancreatic, small intestine, esophageal and colorectal cancer in the Kailuan Cohort study. We prospectively examined the relationship between HBV infection and new‐onset GI cancers among 93 402 participants. Cox proportional hazards regression models, subgroup analyses and competing risk analyses were used to evaluate the association between HBV infection and the risk of new‐onset GI cancers. During a median follow‐up of 13.02 years, 1791 incident GI cancer cases were diagnosed. Compared to HBsAg seronegative participants, a significant positive association between HBV infection and GI cancers was observed in the multivariate‐adjusted models (HR 5.59, 95% CI: 4.84‐6.45). In the site‐specific analyses, participants with HBsAg seropositive exhibited an increased risk of liver cancer (HR = 21.56, 95% CI: 17.32‐26.85), gallbladder or extrahepatic bile duct cancer (HR = 14.89, 95% CI: 10.36‐21.41), colorectal cancer (HR = 1.75, 95% CI: 1.15‐2.96) and pancreatic cancer (HR = 1.86, 95% CI: 1.10‐3.99). After taking death as the competing risk event, the associations of HBV infection with the risk of these cancers were attenuated but remained significant both in the cause‐specific hazards models, the subdistribution proportional hazards models and sensitivity analyses. Our study suggests that HBV infection is associated with the elevated risk of liver cancer and extrahepatic cancer including gallbladder or extrahepatic bile duct, pancreatic and colorectal cancer among adults in Northern China.

## Graphical abstract

What's new?

Both gastrointestinal (GI) cancer and hepatitis B virus (HBV) infection are endemic in China, providing a unique opportunity to investigate suspected associations between HBV and the occurrence of various GI cancers. In this is prospective investigation of data from the Kailuan Cohort study, modeling and analyses indicate that HBV infection is significantly associated with risk of new‐onset GI cancers. Associations were notable particularly for liver cancer and extrahepatic cancers, including cancers of the gallbladder, pancreas and colorectum. The findings suggest that among northern Chinese populations HBV infection has an important role in the progression of cancers of the digestive system.

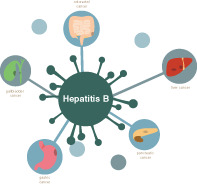

**Abbreviations**
ALT: alanine aminotransferase
BMI: body mass index
cccDNA: covalently closed circular
CIs: confidence intervals
CS model: cause‐specific hazards model
DBP: diastolic blood pressure
GI: gastrointestinal
HBsAg: hepatitis B surface antigen
HBV: hepatitis B virus
HBX: HBV‐encoded X
HCC: hepatocellular carcinoma
HCV: hepatitis C virus
HIV: human immunodeficiency virus
Hp: *Helicobacter pylori*
HRs: hazard ratios
hs‐CRP: high‐sensitivity C‐reactive protein
SBP: systolic blood pressure
SD model: subdistribution proportional hazards model
TC: total cholesterol
TG: triglyceride

## 1 INTRODUCTION

Gastrointestinal (GI) cancer has the highest incidence and mortality among all cancer types.^1^ Due to the short lifespan of its cells, the GI tract has one of the most replicative tissues in the body. Digestive system issues are constantly affected by physical, biological and chemical insults, increasing the risk of oncogenic mutations. Chronic infection with possible carcinogenic agents represents a major risk for subsequent development of cancer and was estimated to be accounted for 2 million incident cancer cases in 2008, with significant variations among regions and countries.^2^ Of these, hepatitis B virus (HBV) infection is a major public health concern. With the implementation of the HBV immunization program in 1984 and the chronic viral hepatitis therapy program in 2003,^3^ its prevalence rate has dropped from 8% to 2%‐7% in the past few years.^4^ However, the Global Hepatitis Report (2017) estimated that nearly 257 million people worldwide were still living with chronic HBV infection,^5^ which would increase the risk of liver fibrosis, liver cirrhosis and hepatocellular carcinoma (HCC) in those infected.^6^, ^7^

The burden of HBV infection, as well as subsequent tissue damage, has been primarily centered in the liver. HBV deposits its life cycle for long‐period persistence in the target tissues by forming a covalently closed circular DNA form (cccDNA) in the nucleus of infected cells.^8^ In animal experiments, HBV DNA has been detected in nonliver tissues including the pancreas, kidney, gonads and lymphoid.^9^, ^10^ Additionally, several clinical case studies revealed that HBV‐specific nucleic acid sequences and related proteins had been detected in extrahepatic tissues of patients with acute or chronic infection of HBV, providing a possible connection of HBV infection to the oncogenesis of extrahepatic cancers.^11^, ^12^ More recently, literature has offered contradictory findings concerning the effect of chronic HBV infection on the occurrence of subsequent nonliver cancers.^13^, ^14^ Despite limited population‐based prospective studies focused on this issue, previous studies may also yield to several limitations including the retrospective design, minimal control of potential confounding factors, short‐term follow‐up, small sample size, hospital‐based identification of individuals and neglect of competing risks events in epidemiologic research, all of which affect the reliability of the existing results.

HBV infection and GI cancers are highly endemic in China, providing a great opportunity to explore the association. The purpose of this investigation is to explore the relationship between chronic HBV infection and the occurrence of GI cancers including liver, gastric, gallbladder or extrahepatic bile duct, pancreatic, small intestine, esophageal and colorectal cancer by drawing data from the Kailuan Cohort study. To further test the validity of the results, stratified analysis and competing risk analysis were also conducted in the current study.

## 2 METHODS

### 2.1 Study population

The data for our study are drawn from the Kailuan Study, which is an ongoing, population‐based, prospective cohort study based on the Kaliuan community in Tangshan City, China.^15^, ^16^ Kailuan Group is a coal industry enterprise that has been involved in many fields such as medical care, education and manufacturing. From July 2006 to October 2007, 155 418 employees (including retirees) from Kailuan Corporation were invited to participate in the physical examinations (the baseline examination) at Kailuan General Hospital and its 10 affiliated hospitals. After obtaining the informed consent, a total of 101 510 participants (65.3%) aged 18 to 98 agreed and were recruited in the study. Face‐to‐face standardized questionnaire surveys, physical examinations, clinical examinations and laboratory tests were conducted for all participants at baseline examination and follow‐up conducted biennially.

**FIGURE 1:**
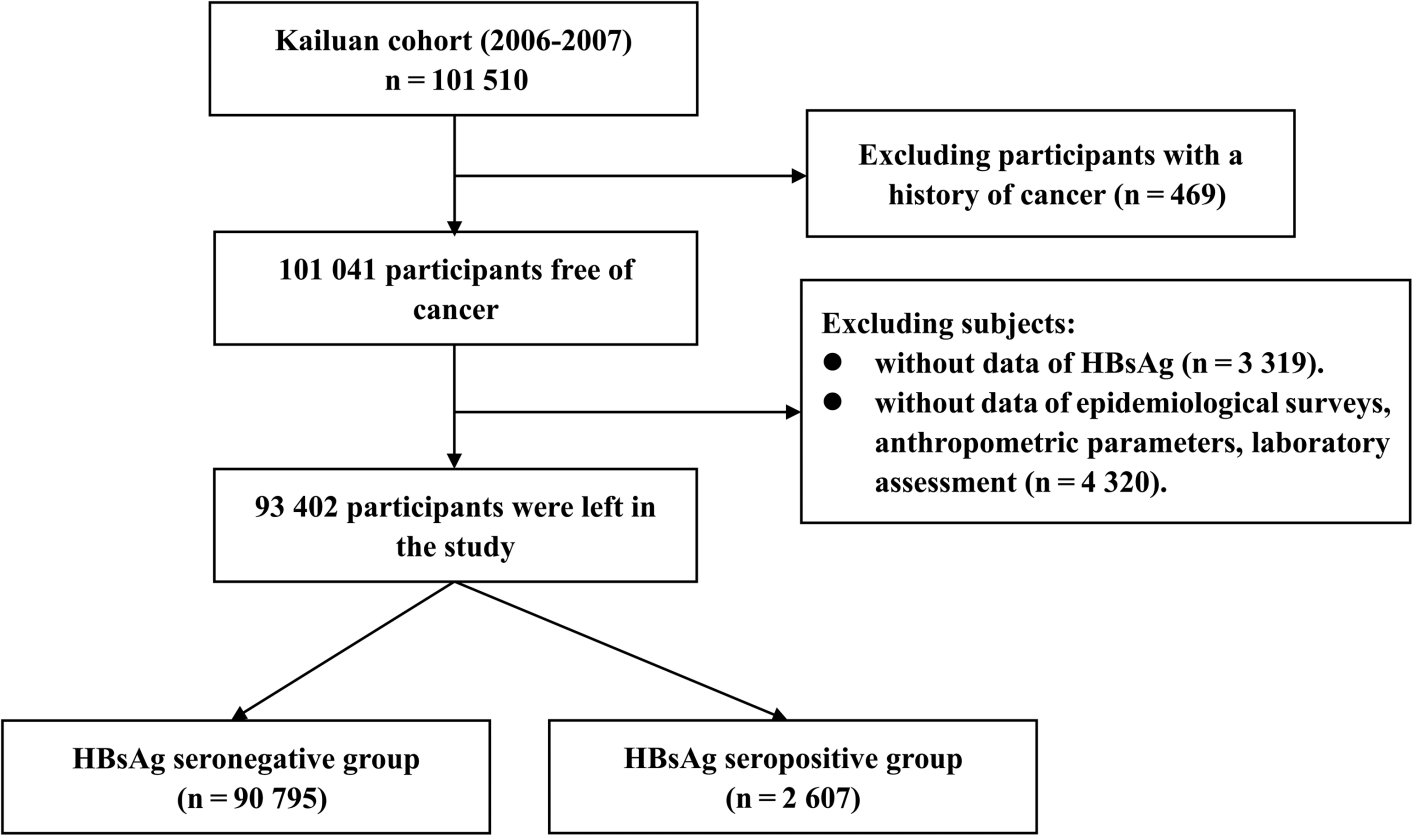
The procedure of participants screening

In our study, participants who did not meet the criteria for analysis were excluded: (a) 469 subjects with a history of malignancy; (b) 2339 subjects with missing or unclear information of hepatitis B surface antigen (HBsAg); (c) 4332 subjects without data of potential confounders including age, sex, body mass index (BMI, in kg/m^2^), high‐sensitivity C‐reactive protein (hs‐CRP, in mg/L), total cholesterol (TC, in mmol/L), alanine aminotransferase (ALT, in u/L), triglyceride (TG, in mmol/L), systolic blood pressure (SBP, in mm Hg), diastolic blood pressure (DBP, in mm Hg), salt consumption, educational background, family income, marital status, smoking status, alcohol drinking, physical exercise, family history of cancer, liver cirrhosis, fatty liver, gallstone disease and gallbladder polyp. There were 93 402 patients (74 637 men and 18 765 women) included in the final analysis (Figure 1). Participants who were excluded were relatively younger (50.85 ± 14.33 years vs 51.51 ± 12.44 years, *P* < .001), with lower levels of BMI (24.82 ± 3.45 vs 25.07 ± 3.49 kg/m^2^, *P* < .001) and exhibited lower infection rate of HBV (179 [2.20%] vs 2598 [2.78%], *P* = .011).

### 2.2 Exposure assessment

After at least 8 hours fast, blood samples were collected to test for HBV infection from each participant at baseline using vacuum tubes containing EDTA. The enzyme‐linked immunosorbent assay was applied to detect hepatitis B surface antigen (HBsAg) quantitatively with a standard operating procedure (Shanghai Kehua Bio‐Engineering, KHB, Shanghai, China). Participants were divided into two groups according to HBV infection status: HBsAg seronegative group (n = 90 795) and HBsAg seropositive group (n = 2607).

### 2.3 Outcome ascertainment

Incident GI cancers were obtained through questionnaires in the routine follow‐up until 31 December 2019. In addition, further cancer cases were identified using medical connections with the provincial vital statistics data, the Tangshan medical insurance system and the Kailuan Social Security Information System annually. Nearly all health information of participants is covered by the Tangshan medical insurance system and the Kailuan social security system. All cancer cases were reconfirmed based on either specific clinical features or positive histopathologic results from the hospitals where patients had received treatment for malignant tumors. When pathological results were unavailable, potential cases were further evaluated by two oncologists. Cancer cases were identified only when two clinicians made the same diagnosis. For patients with multiple tumors and no histopathological results, each tumor was recorded when it was difficult to confirm whether the tumors were the primary tumor or a metastasis. Incident cancer cases were documented according to the International Classification of Diseases, Tenth Revision (ICD‐10): liver (C22.0), gallbladder or extrahepatic bile duct (C23 and C24), gastric (C16), pancreatic (C25), small intestine (C17), esophageal (C15) and colorectal (C18‐C21) cancers.

### 2.4 Potential confounders

Information on age, sex, lifestyle behaviors, educational background, socioeconomic status, medical comorbidities, personal and family medical histories were collected via a standard questionnaire. Current alcohol consumer was defined as having drunk ≥100 mL/day of alcohol lasting for more than 6 months, regardless of the type of alcohol. Current smoker was defined as having 1 cigarette/day at least for more than 6 months. Physical exercise was evaluated from responses regarding the frequency of physical activity (≥3 times/week, ≥30 min/time). Dietary salt intake was self‐reported and classified into three categories: low (<6 g/day), medium (6‐9 g/day) or high (≥10 g/day).

Physical examinations were performed by trained workers for each participant. Height and weight were measured by trained medical staff. BMI was calculated as body weight (kg) divided by the square of height (m^2^) and classified into normal (<24 kg/m^2^), overweight (24.00‐27.99 kg/m^2^) or obese (≥28 kg/m^2^).^17^ Hypertension was defined as: previously diagnosed, and/or an SBP ≥140 mm Hg, and/or a DPB ≥90 mm Hg, and/or using antihypertensive medication.^18^ The ultrasonic examination was used to examine the abdominal region, including liver, gallbladder, pancreas and spleen of each participant after fasting for at least 8 hours by a panel of specialists. Liver cirrhosis, fatty liver, gallstone disease and gallbladder polyp were diagnosed by abdominal ultrasonography according to previous clinically established criteria^19^, ^20^ or through medical records from the Tangshan Medical Insurance System.

All the serum samples were analyzed by an auto‐analyzer (Hitachi 747; Hitachi, Tokyo, Japan) at the central laboratory of Kailuan General Hospital. Serum TC and TG were both measured by the enzymatic colorimetric method (Mind Bioengineering Co. Ltd, Shanghai, China). ALT was measured using an enzymatic rate method (Mind Bioengineering Co. Ltd, Shanghai, China). Hs‐CRP was measured using a high‐sensitivity nephelometry assay (Cias Latex CRP‐H, Kanto Chemical Co. Inc). Serum TBil was measured using a chemical oxidation method (MedicalSystem Biotechnology, China). Diabetes mellitus was defined as fasting blood glucose level ≥ 7.0 mmol/L, and/or taking oral hypoglycemic agents or insulin, and/or a validated physician diagnosis. Hs‐CRP was divided into three groups (<1, 1‐3 and >3 mg/L) based on guidelines from Disease Control and Prevention and the American Heart Association.^21^ Based on the tertiles of each variable, serum TG, TC, ALT and TBil were grouped into three categories.

### 2.5 Statistical analyses

Continuous variables and categorical variables were presented as the mean ± SD and absolute value with percentage. The comparisons of continuous or categorical characteristics were examined using the *t*‐test or *χ* ^2^ test. Person‐years were calculated as the time from baseline examination to the data of cancer diagnosis, death or 31 December 2019, whichever event came first. The Cox proportional hazards regression was used to calculate the hazard ratios (HRs) and their 95% confidence intervals (CIs) for determining the association between HBV infection and cancer development. Three models were fitted as follows: model 1 was an unadjusted analysis; model 2 was adjusted for sex and age (every 10 years); model 3 was further adjusted for BMI, levels of TC, TG, hs‐CRP, TBil and ALT, diabetes, family income, educational background, marital status, salt consumption, smoking status, drinking status, physical exercise and family history of cancer based on model 2. In the pooled GI cancer analyses, we only included the first reported cancer type. However, site‐specific analyses were conducted for all patients with multiple relevant GI cancers. In the site‐specific analyses, model 3 was additionally adjusted for liver cirrhosis and fatty liver in the model of liver cancer, while gallstone disease and gallbladder polyp were further adjusted in the analyses of the gallbladder and biliary cancer. Subgroup analyses were performed for each specific cancer site stratified by sex, age, BMI, smoking status, drinking status, cirrhosis, fatty liver and gallstone disease. The interactions between HBV infection status and these variables were further tested using multiplicative models.

During follow‐up, death may occur before the occurrence of GI cancers. Due to the existence of competing risk events (death), the observation of new‐onset GI cancer cases and further interventions can be hindered. Conventional methods for survival analysis such as standard Cox regression may neglect the competing events and overestimate the risk of the disease. Thus, competing risk analysis should be applied to epidemiologic research. The selection of approach should be determined by the scientific purpose. In general, epidemiological studies focus on two types of issues: (a) Aetiological research is designed to explore the causal relationship between risk factors and an outcome, and applying the cause‐specific hazards (CS) models would be more applicable. (b) Prognostic research is used to predict the probability of the outcome, and applying the subdistribution proportional hazards models (SD) model would be more appropriate. In the current study, CS models and SD models were used to calculate HR_CS_ and HR_SD_ of the specific site of GI cancers with the existence of competing risk, but only if a significant association was found previously in the Cox regressions.

Statistical computations were performed using a commercially available software program (SAS software, version 9.4). Reported *P*‐values are two‐sided, and the significance level was set at *P* < .05.

### 2.6 Sensitivity analyses

Previous studies found patients with liver cirrhosis were associated with an elevated risk of digestive system diseases,^22^, ^23^ therefore, we excluded participants with cirrhosis at baseline and reanalyzed the association to further test the robustness of our findings. Although there was a clear temporal sequencing between HBV exposure and the occurrence of GI cancers, participants who were diagnosed with cancer during the first year of follow‐up were also excluded, because a positive HBV test may lead to additional tests, identifying prevalent cancer. We also estimated an HR for cancers diagnosed within 1 to 3 years, an HR for cancers diagnosed between 3 and 5 years and an HR for cancers detected >5 years after baseline to see how the magnitude of the effect of HBV infection changes over time.

## 3 RESULTS

### 3.1 Characteristics of the study population

Of the 93 402 participants, the mean ± SD age was 51.52 ± 12.43 years with 74 637 (79.91%) males and 18 765 (20.09%) females. The overall age‐ and sex‐standardized HBV infection rate was 3.01% and significantly higher in men (3.10%) than in women (2.02%), which was similar to the previously reported prevalence of HBsAg (<4%) in North China.^24^ The baseline characteristics for participants stratified by HBV infection status are shown in Table 1. Differences in age, sex, TG, TC, ALT, TBil, hs‐CRP, the prevalence of hypertension, physical exercise, current smoker, family income, liver cirrhosis, fatty liver, gallstone disease and gallbladder polyp were found between HBsAg seropositive group and HBsAg seronegative group (*P* < .05). HBsAg seronegative group and HBsAg seropositive group did not differ with respect to BMI, the prevalence of diabetes mellitus, current drinker, family history of cancer, marital status, high salt intake and high‐school graduation (including above).

**TABLE 1:** Baseline characteristics of the participants

	HBsAg seronegative (n = 90 795)	HBsAg seropositive (n = 2607)	*t*/*χ* ^2^	*P* value
Age (year)	51.58 ± 12.45	49.28 ± 11.50	86.87	<.001
Male (%)	72 410 (79.74)	2236 (85.77)	57.35	<.001
TC (mmol/L)			205.98	<.001
<4.51	29 614 (32.61)	1169 (44.84)		
4.51‐5.34	30 503 (33.60)	841 (32.26)		
>5.34	30 678 (33.79)	597 (22.90)		
TG (mmol/L)			122.41	<.001
<1.02	29 617 (32.62)	1057 (40.54)		
1.02‐1.65	30 632 (33.74)	926 (35.52)		
>1.65	30 546 (33.64)	624 (23.94)		
ALT (u/L)			472.96	<.001
<14.9	30 705 (33.82)	484 (18.57)		
14.9‐22.0	28 936 (31.87)	716 (27.46)		
>22.0	31 154 (34.31)	1407 (53.97)		
TBil (μmol/L)			47.19	<.001
<10.7	30 271 (33.34)	715 (27.43)		
10.7‐13.9	30 133 (33.19)	885 (33.95)		
>13.9	30 391 (33.47)	1007 (38.63)		
BMI (kg/m^2^)			0.3678	.832
<24	35 683 (39.30)	1036 (39.74)		
24‐28	38 079 (41.94)	1093 (41.93)		
≥28	17 033 (18.76)	478 (18.34)		
Hs‐CRP (mg/L)			13.32	.001
<1	50 844 (56.17)	1533 (58.89)		
1‐3	23 355 (25.80)	670 (25.74)		
>3	16 319 (18.03)	400 (15.37)		
Physical exercise (%)			15.29	.001
Never	7853 (8.65)	265 (10.16)		
Occasionally	68 574 (75.53)	1989 (76.29)		
Regularly	14 368 (15.82)	353 (13.54)		
Fatty liver (%)			46.56	<.001
None	61 483 (67.93)	1930 (74.20)		
Low grade	18 988 (20.98)	448 (17.22)		
Middle grade	8284 (9.15)	180 (6.92)		
High grade	1749 (1.93)	43 (1.65)		
Current drinker (%)	16 316 (17.97)	455 (17.45)	0.46	.498
Current smoker (%)	28 026 (30.87)	916 (35.14)	21.59	<.001
Family history of cancer (%)	3321 (3.66)	103 (3.95)	1.01	.313
Marital status (married, %)	85 682 (94.37)	2454 (94.13)	0.269	.604
High salt diets (≧10 g/day, %)	9766 (10.76)	301 (11.55)	1.64	.200
High‐school graduation or above (%)	17 878 (19.69)	501 (19.22)	0.359	.549
Reported income of each family member (≧800¥, %)	12 912 (14.22)	338 (12.97)	3.28	.007
Liver cirrhosis (%)	67 (0.07)	109 (4.20)	2276.06	<.001
Gallstone disease (%)	2228 (2.45)	95 (3.64)	14.76	<.001
Gallbladder polyp (%)	729 (0.80)	33 (1.27)	6.70	.010
Hypertension (%)	39 920 (43.97)	1052 (40.35)	13.44	.001
Diabetes mellitus (%)	7640 (8.41)	211 (8.09)	0.339	.560

Abbreviations: ALT, alanine aminotransferase; BMI, body mass index; hs‐CRP, high‐sensitivity C‐reactive protein; TBil, total bilirubin; TC, total cholesterol; TG, triglyceride; WC, waist circumference.

### 3.2 The association of HBV infection with the risk of GI cancers

During a median follow‐up of 13.02 (12.68‐13.20) years, 1791 incident GI cancer cases (colorectal cancer [n = 664], liver cancer [n = 411], gastric cancer [n = 356], pancreatic cancer [n = 167], gallbladder or extrahepatic bile duct cancer [n = 155], esophageal cancer [n = 127] and small‐intestine cancer [n = 9]) were identified, among them 99 (5.53%) participants were diagnosed with multiple tumors. The crude incidence density of GI cancers per 1000 person‐years was 1.57, 1.40 and 7.23 for the total, HBsAg seronegative group and HBsAg seropositive group, respectively. Compared to HBsAg seronegative participants, significant positive associations between HBV infection and the occurrence of pooled GI cancers were observed both in the univariate‐ (HR 5.14, 95% CI: 4.46‐5.92) and multivariate‐adjusted models (HR 5.59, 95% CI: 4.84‐6.45) (Table 2).

**TABLE 2:** The association of HBV infection with the risk of GI cancers

	HBsAg seronegative		HBsAg seropositive		Adjusted hazard ratios (95% CI)
Models	Cases	Person‐years	Cases	Person‐years	
Model 1	1569	1 122 169	222	30 704	5.14 (4.46‐5.92)
Model 2	1569	1 122 169	222	30 704	5.84 (5.06‐6.73)
Model 3	1569	1 122 169	222	30 704	5.59 (4.84‐6.45)

*Note*: Model 1: Univariate analysis. Model 2: Adjusted for age (every 10 years), sex based on model 1. Model 3: Further adjusted for BMI (normal, overweight, obesity), TG, TC, hs‐CRP, TBiL, ALT, diabetes, family income, educational background, marital status, salt consumption, current smoker, drinking status, physical activity and family history of cancer based on model 2.

In the site‐specific analyses, after adjustments were made for the potential confounders, participants with HBsAg seropositive exhibited an increased risk of liver cancer (HR = 21.56, 95% CI: 17.32‐26.85), gallbladder or extrahepatic bile duct cancer (HR = 14.89, 95% CI: 10.36‐21.41), colorectal cancer (HR = 1.75, 95% CI: 1.15‐2.96) and pancreatic cancer (HR = 1.86, 95% CI: 1.10‐3.99) (Table 3). Nonsignificant associations of HBV infection with the risk of esophageal, gastric and small intestine cancer were observed.

**TABLE 3:** The association of HBV infection with the risk of specific site of GI cancer

	HBsAg seronegative		HBsAg seropositive		Adjusted hazard ratios (95% CI)
Cancer type	Cases	Person‐years	Cases	Person‐years	
Liver cancer^a^	236	1 126 629	175	30 894	21.56 (17.32‐26.85)
Gallbladder or extrahepatic bile duct cancer^b^	111	1 127 011	44	31 296	14.89 (10.36‐21.41)
Colorectal cancer	636	1 124 651	29	31 246	1.75 (1.15‐2.96)
Pancreatic cancer	154	1 126 909	13	31 332	1.86 (1.10‐3.99)
Esophageal cancer	124	1 127 007	3	31 354	1.07 (0.34‐3.37)
Stomach cancer	345	1 126 114	11	31 306	1.20 (0.64‐2.25)
Small intestine cancer	9	1 127 239	0	31 356	NA

*Note*: All models were adjusted for age, sex, BMI, TG, TC, hs‐CRP, TBil, ALT, diabetes, family income, educational background, marital status, salt consumption, current smoker, drinking status, physical activity and family history of cancer.

^a^ Further adjusted for liver cirrhosis and fatty liver disease.

^b^ Further adjusted for gallstone disease and gallbladder polyp.

In the competing risk analysis, 9535 participants died before the occurrence of GI cancers during an average follow‐up of 13 years. After taking death as the competing risk event and adjusting for the confounders, similar associations of HBV infection with the risk of liver, gallbladder or extrahepatic bile duct, colorectal and pancreatic cancer were observed both in the CS models and the SD models (Table 4). Because of the null results in the COX regressions, the effects of HBV infection on the risk of esophageal, gastric and small intestine cancer were not explored in the competing risk analysis.

**TABLE 4:** The association of HBV infection with the risk of specific site of GI cancer in competing risk analysis

	HBsAg seronegative		HBsAg seropositive		Adjusted hazard ratios (95% CI)
	Cases	Person‐years	Cases	Person‐years	
CS models					
Liver cancer^a^	236	1 126 629	175	30 894	21.53 (17.30‐26.71)
Gallbladder or extrahepatic bile duct cancer^b^	111	1 127 011	44	31 296	14.88 (10.34‐21.41)
Colorectal cancer	636	1 124 651	29	31 246	1.74 (1.04‐2.94)
Pancreatic cancer	154	1 126 909	13	31 332	1.84 (1.09‐3.60)
SD models					
Liver cancer^a^	236	1 126 629	175	30 894	20.92 (16.68‐26.23)
Gallbladder or extrahepatic bile duct cancer^b^	111	1 127 011	44	31 296	13.66 (9.55‐19.54)
Colorectal cancer	636	1 124 651	29	31 246	1.71 (1.03‐2.91)
Pancreatic cancer	154	1 126 909	13	31 332	1.77 (1.02‐3.51)

*Note*: All models were adjusted for age, sex, BMI, TG, TC, hs‐CRP, TBil, ALT, diabetes, family income, educational background, marital status, salt consumption, current smoker, drinking status, physical activity and family history of cancer.

Abbreviations: CS model, cause‐specific hazard model; SD model, subdistribution hazard function model.

^a^ Further adjusted for liver cirrhosis and fatty liver disease.

^b^ Further adjusted for gallstone disease and gallbladder polyp.

### 3.3 Stratified analysis

**FIGURE 2:**
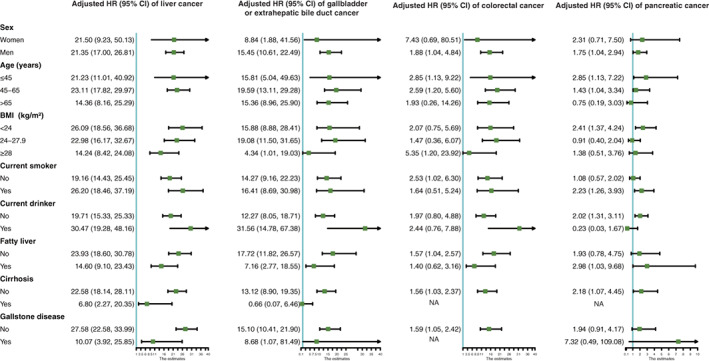
Stratified analysis of the association of HBV infection with the risk of GI cancers. All models were adjusted for age BMI (every 10 years), sex (normal, overweight and obesity), TG, TC, hs‐CRP (<1, 1‐3 and >3 mg/L), TBil, diabetes, family income, educational background, marital status, salt consumption, current smoker, drinking status, physical activity and family history of cancer. Liver cancer models were further adjusted for liver cirrhosis and fatty liver disease. Gallbladder or extrahepatic bile duct cancer models were further adjusted for gallstone disease and gallbladder polyp. (A) Age (every 10 years), BMI (normal, overweight, obesity), current smoker, ALT and drinking status were further adjusted when participants were stratified by gender. (B) Sex, BMI (normal, overweight, obesity), current smoker, ALT and drinking status were further adjusted when participants were stratified by age. Age was also adjusted within each age stratum to prevent residual confounding. (C) Age (every 10 years), sex, ALT, current smoker and drinking status were further adjusted when participants were stratified by BMI. (D) Age (every 10 years), BMI (normal, overweight, obesity), sex, ALT and drinking status were further adjusted when participants were stratified by smoking status. (E) Age (every 10 years), BMI (normal, overweight, obesity), sex and smoking status were further adjusted when participants were stratified by drinking status

Figure 2 showed the stratified analysis by age, gender, BMI, smoking and drinking status. Participants with HBV infection were associated with an elevated risk of liver cancer within all stratified analyses. Smoking and drinking status also showed an effect on the association between HBV infection and the occurrence of liver cancer. Positive associations between HBV infection and gallbladder or extrahepatic bile duct cancer were also observed in all stratified analyses, but none of the tests for interactions were statistically significant. In the analysis of colorectal cancer, significant associations were only observed in those who were male, younger, middle‐aged, with normal BMI, smoking or nondrinker, but not in those who were female, elder, overweight, obese, nonsmoker or current drinker. No interactive effects were revealed within each analysis. In the analysis of pancreatic cancer, significant associations were found only in the participants who were male, young, middle‐aged, obese, nonsmoker and their interactions did not appear to be significant.

### 3.4 Sensitivity analyses

In the sensitivity analysis, after excluding individuals diagnosed with GI cancers within the first year of follow‐up or liver cirrhosis at baseline, the association between HBV infection and the risk of liver, gallbladder or extrahepatic bile duct, colorectal and pancreatic cancer remained significant in the multivariate analysis, the association between HBV infection and the risk of liver, gallbladder or extrahepatic bile duct, colorectal and pancreatic cancer remained significant in the multivariate analysis (Table S1).

Table S2 shows the association of HBV infection with the risk of subsequent GI cancers stratified by the time window of diagnosis. We observed a significant short‐term association (<3 and 3‐5 years) of HBV infection with liver cancer risk, but not for extrahepatic cancer including gallbladder or extrahepatic bile duct, pancreatic or colorectal cancer. In addition, positive association were found for live, gallbladder or extrahepatic bile duct, pancreatic and colorectal cancer diagnosed >5 years after baseline.

## 4 DISCUSSION

In this large population‐based prospective cohort study, we found that participants with chronic HBV infection suffered from a higher risk of GI cancers. Results from the specific‐site analysis showed HBsAg seropositive group was associated with a higher risk of liver cancer and extrahepatic cancer including gallbladder or extrahepatic bile duct, pancreatic and colorectal cancer. Competing risk analysis and sensitivity analyses further validate the robustness of our main findings by considering cancer unrelated‐death or excluding cirrhosis patients. To our knowledge, this is the first study that supports the important role of HBV infection in the progression of carcinogenesis in the digestive system among the northern Chinese.

The association between HBV infection and the risk of gallbladder or extrahepatic bile duct cancer has been previously described. A Korean study by Hong et al reported HBV infected participants were associated with a 1.3‐fold higher risk of gallbladder cancer.^25^ A case‐control study showed a borderline significant association between HBV and extrahepatic bile duct cancer using data of 1 825 316 cases and 200 000 controls from the Surveillance, Epidemiology and End Results (SEER) Medicare database among the elderly within the US population.^26^ Experimental studies have found the presence of HBV DNA in bile duct cancer tissues, indicating the same mechanism as it does for hepatocyte carcinogenesis.^27^, ^28^ A registry‐based, case‐control study by An et al also failed to find a relationship between chronic infection of extrahepatic bile duct cancer and HBV infection.^29^

Several epidemiological studies have shown that HBV infection is closely related to pancreatic cancer. Tian et al found a significant association of HBsAg seropositivity with pancreatic cancer in a case‐control study among the Chinese population.^30^ Song et al found participants who were HBsAg seropositive were associated with an elevated risk of pancreatic cancer in the China Kadoorie Biobank (CKB) prospective cohort study.^31^ In contrast with our observations, null results were also observed in two case‐control studies involving the Korean population and elderly US population.^26^, ^29^ As a case‐control study, the study conducted in South Korea was not suitable for examining the temporal association between potential exposure and the disease, and was more subjected to recall bias than prospective studies. Furthermore, the power in An's study may be limited due to the low HBV infection prevalence in the United States, small sample size, and differences in research design, populations and confounders that were controlled, leading to a null result.^29^ Some studies suggested that HBV infection may play a causal role in the development of colorectal cancer. Kamiza et al determined that there was an increased incidence of colorectal cancer for patients with HBV infection.^32^ Results from a population‐based study in Taiwan showed that participants with HBV infection exhibited a 36% increase in the risk of colorectal cancer compared to HBsAg seronegative participants. Hong et al have reported HBV is associated with a 1.2‐fold higher risk of colorectal cancer.^25^ In contrast to our findings, Mahale et al did not observe an association between HBV infection and risk of colorectal cancer in a case‐control study.

In the current study, we did not find a significant association between HBV infection and gastric cancer. However, the positive association of HBV infection with the risk of gastric cancer has been demonstrated in several previous studies.^25^, ^33^ However, a case‐control study failed to find an association between HBV infection and gastric cancer in the multivariate analysis,^32^ which corroborates our findings. The most recognized factor of gastric cancer is *Helicobacter pylori* (Hp) infection,^34^ which is rarely adjusted because of the limited data in our study as well as most previous studies. This may lead to the discrepancy in the results of different studies.

We provided evidence that HBV infection is closely associated with extrahepatic cancer including gallbladder or extrahepatic bile duct, pancreatic and colorectal cancer and the association is not yielded to analytical methods that consider the competing risk of cancer‐free death. The occurrence of tumors requires long time exposure to risk factors. In our study, 9535 participants died before the occurrence of GI cancers during an average follow‐up of 13 years. The number of deaths far exceeded the number of malignant tumors, which may preclude the observation of GI cancers. Traditional COX regression overestimates the actual risk of the event, and previous studies have encouraged the use of competing risks models in time‐to‐event analyses.^35^, ^36^ Alternative approaches including the CS model and the SD model adopted in the current study. Compared to the results of Cox regression, the risk of developing GI cancers in the competitive risk approaches was slightly reduced. Though results from them were similar, the CS model and SD model are distinct.

A few mechanisms can explain HBV‐induced HCC. However, a limited number of studies have investigated the pathophysiological mechanism of the causality of HBV infection on extrahepatic cancer. HBV is a hepatotropic virus and replicates in hepatocytes.^37^ A previous study found HBV‐encoded X (HBX) protein expression was higher in cancer cells among stomach or pancreatic cancer patients with HBV infection.^31^ As HBV exists in extrahepatic tissue, the role of HBV in the oncogenesis of nonliver cancer is similar to the mechanism of HBV‐induced HCC. The mechanism of HBV‐induced cancer may include direct mechanism and indirect mechanism: (a) Direct mechanism: HBV DNA integrates into the host genome and alters the expression and signaling pathway of the host gene.^38^ (b) Indirect mechanism: The chronic infection of HBV is associated with persistent inflammation, hypoxia, angiogenesis and oxidative stress, all of which may play a role in causing carcinoma.^39^ Recently, the important roles of HBV proteins including HBsAg,^40^ HBeAg^41^ and HBX^42^ induced epigenetic changes have been observed for the expression of genes involved in HCC pathogenesis.

Our study has several strengths. First, it is a large population‐based prospective cohort study, which makes it more efficient for testing a risk factor in the aspect of a direct causal relationship, and is less prone to recall bias than retrospective studies. Second, due to the large sample size and long follow‐up, a good number of incident GI cancer cases were identified which increased the accuracy and creditability of the results. Third, the broad assessments of potential confounders which had been well addressed in our study including cirrhosis, fatty liver, gallstones were not considered in most cohort studies. Fourth, because the Tangshan medical insurance system and the Kailuan social security system contain all the health information of participants, the follow‐up rate was almost 100% in the current study. Last, in the presence of competing risk settings, the implementation of competing risks models makes our results more robust.

Several limitations should also be noted. First, we only use a single measurement of HBsAg. It is likely that there is some degree of variability in HBsAg status over the length of the follow‐up period in a prospective study and may cause a possibility of misclassification. Second, we do not have information on any other HBV marker such as HBeAg and HBV DNA, which hindered us from classifying the population more accurately. Third, specific cancer‐related causal factors including hepatitis C virus (HCV) infection for HCC, and the consumption of cereal, vegetables and high‐fiber food as well as Hp infection for stomach cancer were not collected in our study, which hindered us from assessing the absolute risk of HBV infection more precisely. Though the prevalence of hepatitis C core antibody is only 0.43% in China,^43^ the prevalence of HCV or human immunodeficiency virus (HIV) co‐infection among chronic HBV infected individuals is several times higher compared to people who are not infected with HBV.^44^ The nonexclusion of HCV (and HIV) co‐infected individuals may weaken the results presented in our study. In addition, eating habits are closely related to BMI, TC and TG levels.^45^ Since BMI, TC and TG were adjusted in the multivariate analysis, missing data on dietary habits may have little influence on the results. Results from a systematic review and meta‐analysis showed the pooled Hp prevalence estimate for the general population was 55.8% (95% CI: 51.8‐59.9) in China.^46^ A cross‐sectional study conducted in Tangshan found 2506 (52.25%) were Hp positive among 4796 participants which was line with overall prevalence in China.^47^ Fourth, the industrial nature of the Kailuan community homes mainly labor workers, and there is an imbalance in sex distribution. Nonetheless, we conducted independent statistical research on both genders, thus the impact of imbalance in sex distribution on the results would be minimal. Fifth, the participants were all from Kailuan community and are not nationally representative of the Chinese population. Thus, extrapolated results might not be an accurate description of the wider Chinese population.

## 5 CONCLUSIONS

Our study suggests that HBV infection is associated with the risk of liver cancer, extrahepatic cancers including gallbladder or extrahepatic bile duct, pancreatic and colorectal cancer among adults in Northern China. These findings highlight the importance of early screening for GI cancers in individuals infected with HBV. Future researches need to better assess the existence of HBV DNA and antigens in GI cancers tissues, and elucidate the potential mechanisms of HBV for carcinogenesis.

## CONFLICT OF INTEREST

The authors declare no conflicts of interest.

## AUTHOR CONTRIBUTIONS

All authors have read and approved the manuscript. Tong Liu: Methodology, Software, Writing‐ Original draft preparation. Chunhua Song: Writing‐ Reviewing and Editing. Youcheng Zhang: Reviewing and Editing. Sarah Tan Siyin: Supervision, Validation. Qi Zhang: Writing‐ Reviewing and Editing. Mengmeng Song: Writing‐ Reviewing and Editing. Liying Cao: Conceptualization, Supervision. Hanping Shi: Conceptualization, Supervision, Validation, Resources. The work reported in the article has been performed by the authors, unless clearly specified in the text.

## ETHICS STATEMENT

Our study was approved by the ethics committee of Kailuan General Hospital and followed the Declaration of Helsinki. Informed consent forms were signed by the participants or their legal representatives. Trial registration: Kailuan study, ChiCTR‐TNRC‐11001489. Registered 24 August 2011‐Retrospectively registered, http://www.chictr.org.cn/showprojen.aspx?proj=8050.

## Supporting information


**Table S1** The association of HBV infection with the risk of GI cancer exclude participants who had GI cancer within the first year or with cirrhosis.
**Table S2**. The association of HBV infection with the risk of GI cancer by time window of cancer diagnosis.

## Data Availability

Data will be made available upon reasonable request.
