# Supplementary material for: Hepatitis B virus infection and the risk of gastrointestinal cancers among Chinese population: A prospective cohort study
Source: Int J Cancer. 2021 Dec 14;150(6):1018–28. doi: 10.1002/ijc.33891 (PMC9300134; doi:10.1002/ijc.33891)
Supplement: Supplementary file 1 — Table S1 The association of HBV infection with the risk of GI cancer exclude participants who had GI cancer within the first year or with cirrhosis. Table S2. The association of HBV infection with the risk of GI cancer by time window of cancer diagnosis. [file IJC-150-1018-s001.pdf]

Title: Hepatitis B virus infection and the risk of gastrointestinal cancers among Chinese population: a prospective cohort study

Author list: Tong Liu, Chunhua Song, Youcheng Zhang, Sarah Tan Siyin, Qi Zhang, Mengmeng Song, Liying Cao, Hanping Shi

Supplementary table 1. The association of HBV infection with the risk of GI cancer exclude participants who occurred GI cancer within the 1st year or with cirrhosis.

	HBsAg Seronegative		HBsAg Seropositive		Adjusted Hazard Ratios (95% CI)
	Cases	Person-years	Cases	Person-years	
**Excluding GI cancers occurred within the 1st year**					
Liver cancer [a]	217	1126615	155	30881	21.26(16.93~26.70)
Gallbladder or extrahepatic bile duct cancer [b]	105	1127009	43	31285	12.53(8.45~18.57)
Colorectal cancer	597	1124623	29	31246	1.77(1.16~2.97)
Pancreatic cancer	148	1126905	12	31332	1.82(1.08~3.96)
**Excluding patients with cirrhosis**					
Liver cancer [a]	229	1125971	138	29978	22.99(18.49~28.59)
Gallbladder or extrahepatic bile duct cancer [b]	109	1126340	36	30276	12.92(9.46~20.47)
Colorectal cancer	633	1123979	26	30205	1.71(1.35~2.)
Pancreatic cancer	151	1126237	11	30291	1.72(1.11~3.89)

Note: All models were adjusted for age, sex, BMI, TG, TC, hs-CRP, TBil, ALT, diabetes, family income, educational background, marital status, salt consumption, current smoker, drinking status, physical activity, and family history of cancer.

a: Further adjusted for liver cirrhosis and fatty liver disease.

b: Further adjusted for gallstone disease and gallbladder polyp.

Supplementary table 2. The association of HBV infection with the risk of GI cancer by time window of cancer diagnosis.

	HBsAg Seronegative		HBsAg Seropositive		Adjusted Hazard Ratios (95% CI)
	Cases	Person-years	Cases	Person-years	
**The adjusted HRs (95%CI) for GI cancer diagnosis < 3 years**					
Liver cancer [a]	70	3019	44	156	9.37(5.91~14.86)
Gallbladder or extrahepatic bile duct cancer [b]	17	3074	3	183	2.68(0.57~12.52)
Colorectal cancer	113	3001	2	181	0.38(0.09~1.54)
Pancreatic cancer	33	3057	1	181	0.55(0.07~4.04)
**The adjusted HRs (95%CI) for GI cancer diagnosis within 3-5 years**					
Liver cancer [a]	30	8044	31	341	29.66(16.84~52.22)
Gallbladder or extrahepatic bile duct cancer [b]	8	8061	0	377	NA
Colorectal cancer	110	7091	3	373	0.66(0.21~2.11)
Pancreatic cancer	13	8052	1	370	1.94(0.24~15.39)
**The adjusted HRs (95%CI) for GI cancer diagnosis > 5 years**					
Liver cancer [a]	136	1115566	100	30397	24.39(18.44~32.27)
Gallbladder or extrahepatic bile duct cancer [b]	86	1115876	41	30736	19.99(13.72~29.11)

**Colorectal cancer**	**413**	**1114559**	**24**	**30692**	**1.80(1.12~2.89)**
**Pancreatic cancer**	**108**	**1115800**	**11**	**30781**	**2.03(1.09~3.67)**

Note: All models were adjusted for age, sex, BMI, TG, TC, hs-CRP, TBil, ALT, diabetes, family income, educational background, marital status, salt consumption, current smoker, drinking status, physical activity, and family history of cancer.

a: Further adjusted for liver cirrhosis and fatty liver disease.

b: Further adjusted for gallstone disease and gallbladder polyp.
